# Multiparametric Ultrasound Findings of Testicular Sarcoidosis

**DOI:** 10.1002/jum.70197

**Published:** 2026-02-06

**Authors:** Huahui Liu, Razan Abudalo, Ghali Salahia, Gibran T. Yusuf, Dean Y. Huang, Paul S. Sidhu

**Affiliations:** ^1^ Department of Radiology King's College Hospital London UK; ^2^ Department of Medical Ultrasonics The Eighth Affiliated Hospital of Sun Yat‐Sen University Shenzhen China; ^3^ Department of Radiology Jordian Royal Medical Services Amman Jordan; ^4^ Department of Clinical Radiology University Hospital of Wales Cardiff UK; ^5^ Department of Imaging Sciences, School of Biomedical Engineering and Imaging Sciences, Faculty of Life Sciences and Medicine King's College London London UK

**Keywords:** contrast‐enhanced ultrasound, multiparametric ultrasound, strain elastography, testicular sarcoidosis

## Abstract

**Objectives:**

To characterize the multiparametric ultrasound (MPUS) features of testicular sarcoidosis, incorporating greyscale, color Doppler ultrasound (CDUS), contrast‐enhanced ultrasound (CEUS), and strain elastography (SE), and to assess their collective diagnostic value.

**Methods:**

A retrospective review of our institutional ultrasound database identified patients with testicular lesions and a confirmed diagnosis of sarcoidosis (via histopathology or established clinical criteria) between May 2009 and June 2025. All patients underwent a standardized scrotal MPUS protocol. Lesion characteristics on greyscale, vascularity on CDUS and CEUS, and tissue stiffness on SE were systematically analyzed.

**Results:**

Seventeen patients (mean age: 42.1 ± 12.4 years) were included. The MPUS pattern was consistent. On grayscale, all lesions were small, solid (mean largest diameter: 4.3 ± 1.8 mm), hypoechoic, and well‐defined. Lesions were multifocal (76.5%) and bilateral (52.9%). CDUS showed absent (47.1%) or low (35.3%) internal vascularity. CEUS demonstrated no enhancement in 62.5% of lesions. SE indicated intermediate (53.3%) or soft (26.7%) tissue elasticity. Follow‐up ultrasound demonstrated temporal stability in 76.5% of lesions.

**Conclusion:**

Testicular sarcoidosis exhibited a suggestive MPUS signature characterized by small, hypoechoic, solid, hypovascular, and stable lesions, often with soft or intermediate elasticity. Recognition of this pattern in the appropriate clinical context can strongly suggest this rare benign diagnosis, guiding conservative management and preventing unnecessary orchidectomy.

**Abbreviations**
CDUS: color Doppler ultrasound
CEUS: contrast‐enhanced ultrasound
MPUS: multiparametric ultrasound
SE: strain elastography

## Introduction

Granulomatous diseases encompass a wide range of conditions, unified by a histological finding of granuloma formation.^1^ Histologically this is seen as mononuclear inflammatory cells surrounded by lymphocytes. Most frequently granulomatous disease is a multisystem disorder but may present in a localized pattern and is dependent on the underlying etiology, for example, autoimmune, infectious, idiopathic and hereditary.^2^

Radiologic findings of malignancy potentially have considerable overlap with findings of granulomatous disease^3^ and without prior clinical diagnosis, laboratory results, or suggestive symptoms and signs, differentiation between the 2 may be difficult. Differentiation is critical, given the benign course in granulomatous disease and often conservative management, while testicular malignancy usually requires expedited surgery.^4^

Sarcoidosis is a chronic non‐caseating granulomatous disease of unknown etiology, which can affect any part of the body. Most cases usually involve the chest, with the urogenital system only involved in 0.2%.^5^ For those with genital tract involvement, the epididymis is involved in 73% and 43% involving the testes.^6^ Testicular sarcoidosis is particularly rare with only 60 described cases.^7^ The clinical presentation of testicular sarcoidosis is nonspecific and varies from a diffuse painless scrotal mass to testicular pain and swelling.^8^ The clinical presentation may mimic both a testicular tumor and acute epididymo‐orchitis.^9^ Both sarcoidosis and testicular neoplasia occur in the same age group (20–40 years) and differentiating the 2 is paramount to avoid unnecessary orchidectomy in an age group where fertility is of particular importance.

Ultrasound, as the primary method to assess the scrotal contents, plays a vital role. Combining all aspects of the ultrasound examination, termed multiparametric ultrasound (MPUS),^10^ is likely to improve the diagnostic confidence in the assessment of the intra‐testicular lesion, previously applied with other neoplastic intra‐testicular lesions.^11^ There is a single description using MPUS features for the assessment of a case of testicular sarcoidosis, but there is a paucity of evidence of the use of MPUS for aiding the characterization of testicular sarcoidosis.^12^

The primary objective of this study was to characterize the greyscale, color Doppler ultrasound (CDUS), contrast‐enhanced ultrasound (CEUS) and strain elastography (SE) features of this entity to establish a recognizable imaging pattern.

## Materials and Methods

### Study Design and Patient Selection

This retrospective, single‐center case series was conducted after approval from the local Institutional Review Board. All patients verbally consented to the ultrasound examination, including greyscale, CDUS, CEUS and SE, which have been standard practice at our institution for evaluation of focal testicular abnormalities from 2009. A comprehensive search of our departmental radiology information system and ultrasound database was performed to identify all patients who underwent scrotal ultrasound for the evaluation of focal testicular lesions between May 2009 and June 2025.

Patients were included if they met the following diagnostic criteria for testicular sarcoidosis: (1) histopathological confirmation from testicular biopsy demonstrating non‐caseating granulomas, consistent with sarcoidosis, (2) histopathological confirmation from extra‐testicular (e.g., lung, lymph node, skin) biopsy demonstrating non‐caseating granulomas, consistent with sarcoidosis, and/or (3) a well‐established clinical diagnosis of systemic sarcoidosis with compatible testicular lesions on imaging, supported by negative tumor markers (alpha‐fetoprotein and beta‐human chorionic gonadotropin) and the absence of clinical or radiological evidence of other malignancies. Patients with a history of testicular trauma, active infection, or known primary testicular or hematological malignancy were excluded.

A total of 17 patients fulfilled the inclusion criteria and constituted the final study cohort.

### Ultrasound Examination Protocol

All patients underwent a standardized MPUS examination of the scrotum, performed by 1 of 3 experienced radiologists (each with >10 years of expertise in scrotal ultrasound, CEUS and elastography techniques). Examinations were conducted using high‐end ultrasound systems (Acuson S3000 [Siemens Healthineers, Erlangen, Germany]; Samsung RS80A [Samsung Medison, Seoul, Korea]; Hitachi Arietta 60 [Hitachi Medical Corporation, Tokyo, Japan]; Logiq E9 [GE Healthcare, USA]) equipped with high‐frequency linear array transducers (transducers labelled 9L4, 14L5, L65, 9L, respectively).

The MPUS protocol systematically acquired the following sequences for each identified lesion:

Greyscale Ultrasound: To assess location, size (maximum diameter), shape, margin characteristics (smooth or irregular), and echogenicity relative to the surrounding normal testicular parenchyma.

Color Doppler Ultrasound: To evaluate intralesional vascularity. Settings (pulse repetition frequency, gain, wall filter) were optimized to maximize sensitivity for low‐velocity flow without introducing artifacts. Vascularity was categorized subjectively as avascular, hypo‐vascular, or hyper‐vascular.

Strain Elastography: Performed using a dedicated ultrasound system (HV900, Hitachi, Japan) with a 14–6 MHz linear transducer and real‐time tissue elastography (RTE®) software. The technique involved applying light, repetitive compression with the transducer. The system displayed a color‐coded elastogram superimposed on the B‐mode image, with a scale from red (softest) to blue (hardest). For semi‐quantitative analysis, a strain ratio was calculated by comparing the elasticity of the target lesion to a reference region of normal testicular parenchyma. An elasticity score (1: soft to 6: hard) was also assigned based on the color pattern, with scores of 1–2 classified as soft, 3–4 as intermediate, and 5–6 as hard, based on established grading criteria.^13^, ^14^

Contrast‐Enhanced Ultrasound: Performed after a bolus intravenous injection of 4.8 mL of the sulfur hexafluoride microbubble contrast agent (SonoVue®, Bracco Imaging, Milan, Italy). A low mechanical index (<0.1) was used to minimize bubble destruction. Dynamic cine loops were recorded continuously for at least 90 seconds post‐injection. Enhancement patterns were classified as no enhancement, iso‐enhancement, or hyper‐enhancement relative to the adjacent testicular parenchyma.

### Image Analysis and Data Collection

All stored ultrasound images and cine loops were retrospectively reviewed in consensus by 2 experienced radiologists who were aware of the final diagnosis (DYH 20 years' experience, GTY 15 years' experience). For each patient, the reviewers recorded the qualitative and quantitative MPUS features on a standardized data collection form. In cases of multiple lesions, the largest or most representative lesion was selected for detailed analysis. Stability was confirmed by reviewing all available follow‐up ultrasound examinations and documenting any change in lesion size or characteristics. Any disagreements were subject to further evaluation by a third observer (PSS, 30 years' experience).

### Statistical Analysis

Descriptive statistics were used to summarize the data. Continuous variables (e.g., age, lesion size) are presented as mean ± standard deviation and range. Categorical variables (e.g., echogenicity, vascularity patterns) are presented as frequencies and percentages. All analyses were performed using standard statistical software (IBM SPSS Statistics, Version 26.0).

## Results

### Basic Data and Imaging Characteristics

A total of 17 male patients with a diagnosis of testicular sarcoidosis were included in this retrospective study. The mean age was 43.1 ± 12.4 years (range, 27–64 years). Lesions were multifocal in 13 patients (76.5%), bilateral in 9 patients (52.9%) and unilateral in 8 patients (47.1%). The mean largest lesion diameter was 4.3 ± 1.8 mm (range, 2.0–9.0 mm). The diagnosis was established on histology via testicular biopsy in 3 patients (17.6%) and by biopsy of other affected organs in 9 patients (52.9%). In the remaining 5 patients (29.4%), the diagnosis was based on a comprehensive clinical assessment in conjunction with typical imaging findings and stable follow‐up. Patient demographics, lesion characteristics, and diagnostic pathways are detailed in Table 1.

**Table 1:** The Patient's Basic Data and Imaging Characteristics

Number	Age	Lesion Location	Largest Size (mm)	Grey Scale Ultrasound	Color Doppler Lesion Vascularity	Contrast‐Enhanced Lesion Vascularity	Elastography Appearance	How Was the Diagnosis Established?	Sonographic Follow‐Up
1	27	Bilateral multifocal	4	Hypoechoic	Hypo‐vascular	Iso‐enhancement	Intermediate	Testicular biopsy	Increased in number and size
2	40	Bilateral multifocal	4	Hypoechoic	Avascular	No enhancement	Soft	Testicular biopsies	Stable appearance
3	28	Left single	6.7	Hypoechoic	Hypo‐vascular	Iso‐enhancement	Intermediate	Testicular biopsy	Decreased in number and size
4	28	Bilateral multifocal	2	Hypoechoic	Avascular	No enhancement	‐	Biopsies in other organs	Stable appearance
5	40	Left single	3	Hypoechoic	Hypo‐vascular	No enhancement	Intermediate	Biopsies in other organs	Stable appearance
6	35	Bilateral multifocal	4	Hypoechoic	Hypovascular	Hyperenhancement	Intermediate	Biopsies in other organs	Stable appearance
7	39	Right multifocal	5	Hypoechoic	Peripheral vascularity	Iso‐enhancement	Hard	Biopsies in other organs	Decreased in number and size
8	34	Right multifocal	3	Hypoechoic	Slightly hyper‐vascular	Iso‐enhancement	Hard	Biopsies in other organs	Stable appearance
9	45	Left multifocal	4	Hypoechoic	Avascular	No enhancement	‐	Biopsies in other organs	Stable appearance
10	38	Bilateral multifocal	3	Hypoechoic	Avascular	No enhancement	Intermediate	Biopsies in other organs	Stable appearance
11	62	Bilateral multifocal	4	Hypoechoic	Avascular	No enhancement	Intermediate	Biopsies in other organs	Stable appearance
12	36	Bilateral multifocal	4	Hypoechoic	Avascular	No enhancement	Hard	Biopsies in other organs	Stable appearance
13	64	Bilateral multifocal	4	Hypoechoic	Hypo‐vascular	Hyperenhancement	Intermediate	Clinical comprehensive diagnosis	Stable appearance
14	60	Left multifocal	3	Hypoechoic	Hypo‐vascular	‐	Soft	Clinical comprehensive diagnosis	Decreased in number and size
15	32	Bilateral multifocal	3	Hypoechoic	Avascular	No enhancement	Soft	Clinical comprehensive diagnosis	Stable appearance
16	59	Left single	9	Hypoechoic	Avascular	No enhancement	Intermediate	Clinical comprehensive diagnosis	Stable appearance
17	49	Right single	7	Hypoechoic	Slightly hyper‐vascular	No enhancement	Soft	Clinical comprehensive diagnosis	Stable appearance

### Multiparametric Ultrasound Findings

The observers agreed with the findings without need for further evaluation. The MPUS features across the cases demonstrated remarkable consistency (Table 2). On grayscale ultrasound, all 17 lesions (100%) were uniformly hypoechoic and entirely solid. All lesions (100%) exhibited a circular or elliptical shape, with smooth margins in 15 cases (88.2%) and irregular margins in 2 cases (11.8%).

**Table 2:** Multiparametric Ultrasound Findings

Parameters	Value	%
Echogenicity		
Hypoechoic	17	100
Shape		
Circular or elliptical	17	100
Margin		
Smooth	15	88.2
Irregular	2	11.8
Composition		
Solid totally	17	100
Mixed cystic and solid	0	
Size		
Mean (mm)	4.3 ± 1.8	‐
Range (mm)	2.0–9.0	‐
Number		
Multifocal	13	76.5
Single	4	23.5
Lesion location		
Bilaterally	9	52.9
Unilaterally	8	47.1
Color Doppler lesion vascularity		
Avascular	8	47.1
Hypo‐vascular	6	35.3
Slightly hyper‐vascular	2	11.8
Peripheral vascularity	1	5.9
Contrast‐enhanced lesion vascularity (n = 16)		
No enhancement	10	62.5
Iso‐enhancement	4	25.0
Hyperenhancement	2	12.5
Elasticity Score (n = 15)		
1–2 (soft)	4	26.7
3–4 (intermediate)	8	53.3
5–6 (hard)	3	20.0
Sonographic follow‐up		
Increased in number and size	1	5.9
Stable appearance	13	76.5
Decreased in number and size	3	17.6

Color Doppler ultrasound revealed predominantly absent or low internal vascularity. Lesions were avascular in 8 cases (47.1%) and hypo‐vascular in 6 cases (35.3%). Two lesions (11.8%) showed slight hypervascularity, and 1 lesion (5.9%) demonstrated only peripheral vascularity.

Strain elastography was performed in 15 patients. Most lesions (8 lesions, 53.3%) displayed an intermediate elasticity score (3–4). Four lesions (26.7%) were categorized as soft (scores 1–2), and 3 lesions (20.0%) were hard (scores 5–6).

Contrast‐enhanced ultrasound findings were available for 16 lesions. The majority (10 lessons, 62.5%) demonstrated no internal contrast enhancement. Iso‐enhancement was observed in 4 lesions (25.0%), and hyperenhancement was seen in 2 lesions (12.5%).

**Figure 1:**
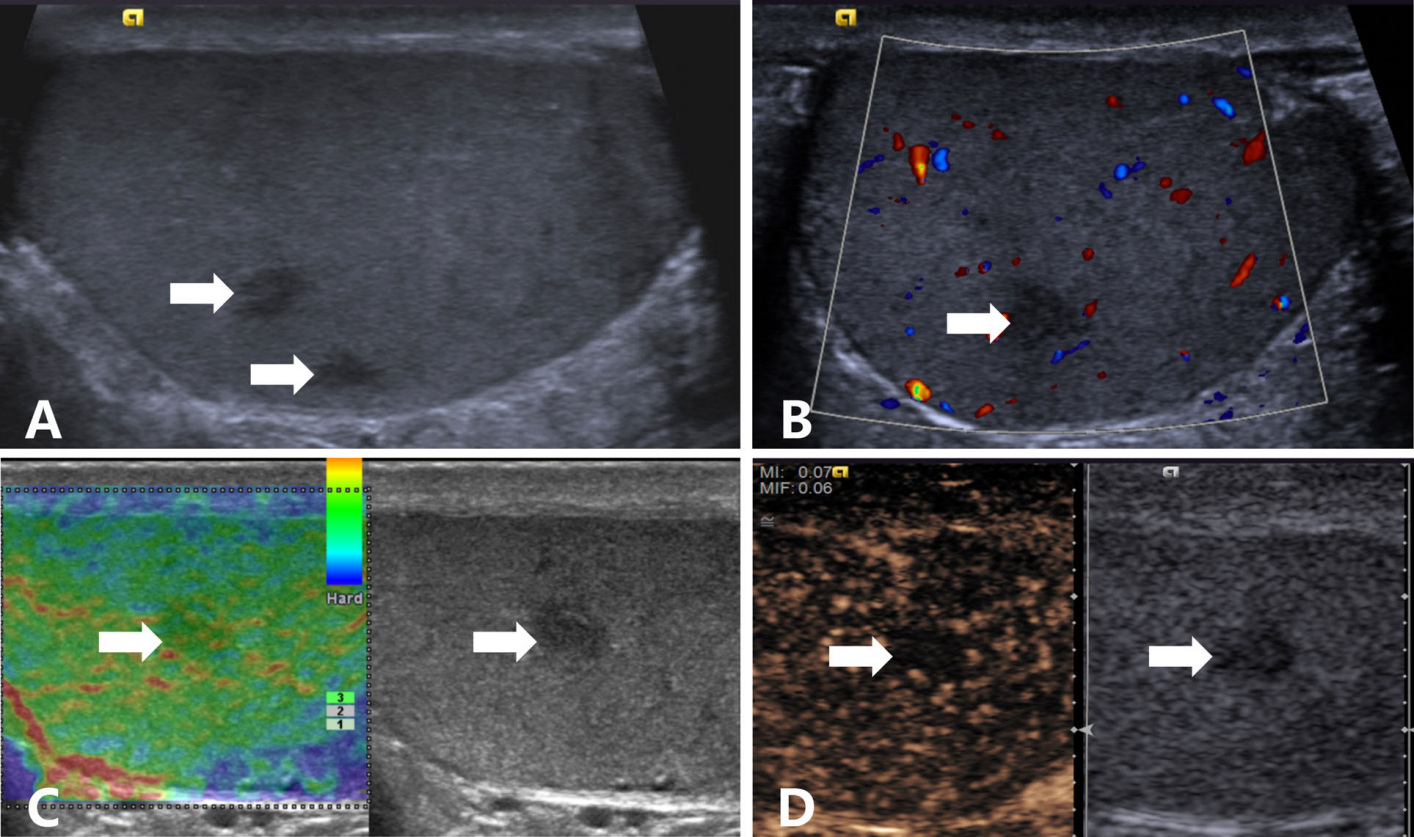
A 40‐year‐old male with multifocal small areas of decreased reflectivity in both testes on ultrasound surveillance. **A**, Greyscale ultrasound of both testes showed hypo‐echoic multifocal lesions (the largest size: 5 mm, arrows). **B**, Color Doppler ultrasound (CDUS) image demonstrates avascularity within the lesions (arrow). **C**, Strain elastography (SE) showed a lesion with moderate stiffness (arrows). **D**, Contrast‐enhanced ultrasound (CEUS) revealed no enhancement (arrow). The histology from a biopsy showed testicular sarcoidosis.

**Figure 2:**
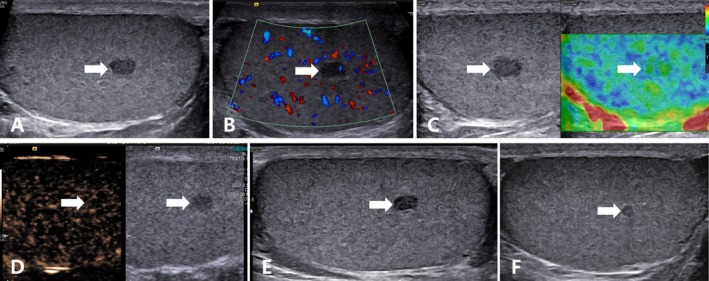
A 39‐year‐old male who was known to have pulmonary sarcoidosis attended the urology clinic complaining of intermittent right‐sided dull testicular ache. **A**, Greyscale ultrasound of the right testis showed hypoechoic lesions (the largest measuring 4 mm, arrow). **B**, Color Doppler ultrasound (CDUS) image demonstrated avascularity within the lesions (arrow). **C**, Strain elastography (SE) showed a lesion with moderate stiffness (arrows). **D**, Contrast‐enhanced ultrasound (CEUS) revealed iso‐enhancement (arrows). **E**, An ultrasound follow‐up showed stable appearances at 3 months (arrow). **F**, An ultrasound follow‐up showed a decrease in number and size (the largest size: 2 mm, arrow) at 2 years.

All patients underwent follow‐up ultrasound examinations ranging from 6 months to 10 years (median 3 years). Most testicular lesions (76.5%) demonstrated temporal stability in size and imaging characteristics on serial scans (Figure 1); 1 of the lesions exhibited slightly interval growth and changes in number and size, but no changes in echogenicity or vascular patterns during follow‐up at 1 year, with testicular biopsy showing granuloma consistent with sarcoidosis; 2 of the lesions exhibited significant decreased in number and size (Figure 2).

## Discussion

This study delineated the MPUS characteristics of testicular sarcoidosis in a cohort of 17 patients. The results consistently demonstrated lesions as small, circumscribed, solid, and hypoechoic nodules. Vascular assessment revealed predominantly absent or minimal internal flow on CDUS, a finding corroborated on CEUS, where the majority of lesions (62.5%) exhibited no enhancement. Strain elastography predominantly showed intermediate (53.3%) to soft (26.7%) tissue stiffness. Notably, in our series, the majority of lesions (76.5%) demonstrated stability over a median follow‐up of 3 years. This relative stability, even in patients on varying treatment regimens for systemic disease, may reflect the chronic, fibrotic nature of established granulomas. This constellation of features forms a distinct, though not entirely pathognomonic, MPUS profile.

The imaging features can be interpreted within the known histopathology of sarcoidosis. The uniform hypo‐echogenicity likely corresponds to the homogeneous cellular composition of compact, non‐caseating granulomas, which present few acoustic interfaces compared to the surrounding testicular parenchyma.^15^ The predominant hypo‐vascularity or avascularity on CDUS is consistent with the relatively acellular, fibrotic core of established granulomas, which possess a sparse microvascular network compared to the hyper‐vascular stroma typical of most malignant germ cell tumors.^16^ The addition of CEUS is known to improve the diagnostic confidence in the interpretation of the presence of vascularity within the lesion, potentially differentiating neoplastic from non‐neoplastic lesions.^17^ It is crucial to note that the absence of internal enhancement on CEUS strongly supports a benign, often non‐viable or acellular process (e.g., infarction, old hematoma, or fibrotic core), whereas the presence of enhancement—whether iso‐ or hyper‐enhancement, as observed in a minority of our cases (37.5%)—is non‐specific and can be seen in active inflammatory, granulomatous, and neoplastic processes.^18^ Therefore, CEUS findings must not be interpreted in isolation.

The tendency toward intermediate or soft elasticity on SE aligns with the mechanical properties of cellular inflammatory aggregates, which are generally less stiff than the dense, desmoplastic stroma often associated with carcinomas. These findings differ from typical testicular malignancies, which are more often hyper‐vascular, may exhibit cystic or heterogeneous components, and can be stiff on elastography.^19^

In this diagnostic context, MPUS moves beyond the limitations of any single modality, providing a synergistic diagnostic framework. While greyscale ultrasound raises the initial suspicion, it lacks specificity.^14^ The addition of CEUS critically evaluates micro‐perfusion, effectively distinguishing the hypo‐enhancing granulomas from typically hyper‐enhancing tumors.^20^ Elastography adds a complementary biomechanical dimension, further supporting a non‐desmoplastic, inflammatory etiology.^21^ Therefore, MPUS does not merely describe features in parallel but integrates them to create a composite “imaging biomarker” with higher diagnostic confidence than any individual parameter. The imaging pattern described, while consistent in our cohort, is not pathognomonic. This integrated approach is pivotal for suggesting a benign diagnosis like sarcoidosis in a clinical setting where malignancy is the primary concern, thereby guiding management toward surveillance or biopsy rather than immediate radical surgery.

Its diagnostic value is highest when interpreted within the appropriate clinical context: a patient with known systemic sarcoidosis, negative tumor markers (AFP, β‐hCG), and absence of systemic symptoms suggestive of active infection or lymphoma. Even with suggestive MPUS features, management should be individualized. For lesions demonstrating any internal vascularity on CEUS (iso‐ or hyper‐enhancement) or in cases without an established systemic diagnosis, short‐term imaging follow‐up (e.g., 3–6 months) is a prudent and widely accepted strategy for small, non‐palpable intratesticular lesions to document stability, which strongly supports a benign course.^22^

This study has several limitations. Its retrospective nature and the small sample size, inherent to the rarity of the condition, limit the statistical power and generalizability of the findings. The diagnostic standard was heterogeneous; only a minority of cases (17.6%) were confirmed by direct testicular histology, with most diagnoses relying on extra‐testicular biopsy or clinical criteria, although this reflects real‐world practice where orchidectomy is avoided. Strain elastography remains operator‐dependent, introducing potential variability.^23^ Finally, the variable follow‐up findings (stability vs. change) highlight that the MPUS pattern, while characteristic, does not equate to absolute biological inertness and must be interpreted within the broader clinical and serial imaging context.

## Conclusion

In conclusion, testicular sarcoidosis exhibits a suggestive MPUS signature most commonly characterized by small, solid, hypo‐echoic, hypo‐vascular lesions with intermediate/soft elasticity and a strong tendency toward stability on follow‐up. This pattern, rooted in the underlying granulomatous pathology, differs meaningfully from that of common testicular malignancies. The integration of multiple ultrasound parameters significantly enhances diagnostic specificity compared to conventional ultrasound alone. Recognition of this MPUS profile, in conjunction with pertinent clinical and serological data, can support a conservative diagnostic pathway—favoring surveillance or targeted biopsy—thereby aiding in the prevention of unnecessary orchidectomy and preserving testicular function.

## Data Availability

The data that support the findings of this study are available on request from the corresponding author. The data are not publicly available due to privacy or ethical restrictions.
